# Hepatoprotective Effects of Albumin-Encapsulated Nanoparticles of a Curcumin Derivative COP-22 against Lipopolysaccharide/D-Galactosamine-Induced Acute Liver Injury in Mice

**DOI:** 10.3390/ijms23094903

**Published:** 2022-04-28

**Authors:** Wenwen Mu, Qi Wang, Mingxia Jia, Sijia Dong, Sijie Li, Jie Yang, Guoyun Liu

**Affiliations:** School of Pharmaceutical Sciences, Liaocheng University, 1 Hunan Street, Liaocheng 252059, China; wenwenmu1115@gmail.com (W.M.); wangqi010709@gmail.com (Q.W.); j2019207670@gmail.com (M.J.); dongsijia1104@gmail.com (S.D.); lisijie1910@gmail.com (S.L.)

**Keywords:** acute liver injury, bovine serum albumin nanoparticles, curcumin derivative, inflammation, oxidative stress, apoptosis

## Abstract

Acute liver injury (ALI) is a severe syndrome and can further develop into acute liver failure (ALF) which can lead to high mortality and cause irreversible liver injuries in the clinic. Liver transplantation is the most common treatment; however, liver donors are lacking, and the progression of ALF is rapid. Nanoparticles can increase the bioavailability and the targeted accumulation of drugs in the liver, so as to significantly improve the therapeutic effect of ALI. Curcumin derivative COP-22 exhibits low cytotoxicity and effective anti-inflammatory activity; however, it has poor water solubility. In this study, COP-22-loaded bovine serum albumin (BSA) nanoparticles (22 NPs) were prepared and characterized. They exhibit effective hepatoprotective effects by inhibiting inflammation, oxidative stress, and apoptosis on Lipopolysaccharide/D-Galactosamine-induced acute liver injury of mice. The anti-inflammatory activity of 22 NPs is related to the regulation of the NF-κB signaling pathways; the antioxidant activity is related to the regulation of the Nrf2 signaling pathways; and the apoptosis activity is related to mitochondrial pathways, involving Bcl-2 family and Caspase-3 protein. These three cellular pathways are interrelated and affected each other. Moreover, 22 NPs could be passively targeted to accumulate in the liver through the retention effect and are more easily absorbed than 22.HCl salt in the liver.

## 1. Introduction

The liver is an important metabolic organ in vertebrates. It plays a key role in the host’s defensive response, which can scavenge pathogenic microorganisms and toxins [1]. Meanwhile, it is also the victim of these attacks, which can result in the activation of host immune cells and then incite inflammation [2]. Acute liver injury (ALI) is a severe syndrome, which can be induced by many risk factors, such as infections, drugs, toxins, etc. [3]. ALI can further develop into acute liver failure (ALF) which can lead to high mortality and cause irreversible liver injuries in the clinic [4]. Liver transplantation is the most common treatment which is proven to be effective for irreversible ALF [5,6].

However, liver donors are lacking, and the progression of ALF is rapid. Hence, it is a great challenge to find more efficient therapeutic treatments for ALI and to interdict the progression of acute liver injury into acute hepatic failure [7].

The lipopolysaccharide (LPS)/D-galactosamine (D-GalN)-induced acute liver injury in mice has been widely used to screen for potential hepatoprotective agents [8,9,10]. LPS is a component of the outer membrane of Gram-negative bacteria and a representative stimulator of inflammation. It can activate Kupffer cells, a specialized population of macrophages that reside in the liver, resulting in the production of high levels of pro-inflammatory cytokines, such as TNF-α, IL-1, and IL-6 [11,12]. However, LPS is less specific to liver damage. D-GalN can induce a selective transcriptional block in hepatocytes and can act as a sensitizing agent in LPS-induced liver injury [13,14]. This model is similar to liver injury in clinical settings. Thus far, studies have shown that the molecular mechanisms of ALI are related to inflammation, oxidative stress, and apoptosis [15,16].

**Figure 1:**
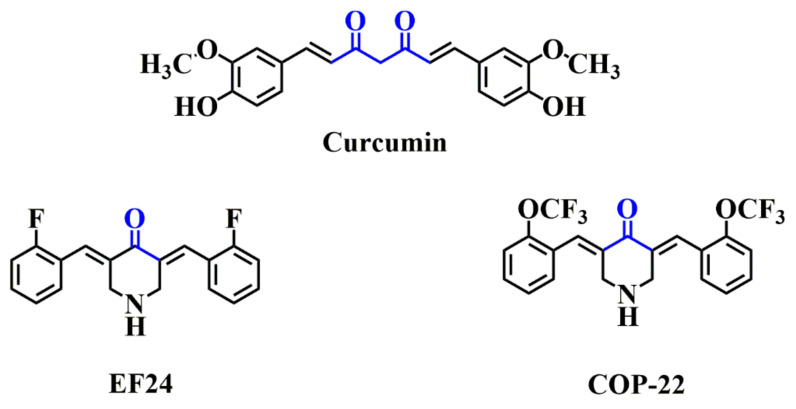
Chemical structures of curcumin, EF24, and COP-22.

Natural products are the inspiration for drug discovery, and structural modifications of natural products are an important strategy in pharmaceutical discovery. Curcumin is the main pharmacologically active curcuminoid pigment in turmeric. It has a variety of biological activities, including anti-cancer, anti-inflammatory, anti-oxidant, immunomodulatory effects, etc. [17]. However, curcumin has relatively low bioavailability due to its easily decomposable α-diketone moiety [18,19]. In recent years, many mono-carbonyl curcumin derivatives have been developed with improved biological activities in preventing and treating various diseases [20,21,22,23]. For example, a fluorinated substance EF24 [22] (3,5-bis((E)-2-fluorobenzylidene)piperidin-4-one, Figure 1) exerted its anti-cancer activity by inhibiting NF-κB, inhibiting HIF-1α activity, regulating reactive oxygen species, etc.; moreover, EF24 showed promising anti-inflammatory and anti-microbial activities. Another mono-carbonyl curcumin derivative COP-22 [24] (3,5-bis((E)-2-(trifluoromethoxy)benzylidene)piperidin-4-one, Figure 1), exhibited low cytotoxicity, and was shown to inhibit LPS-induced expression of pro-inflammatory cytokine, the production of oxidative stress, and NF-κB nuclear translocation in Raw264.7 cells. Additionally, the inhibitory activity of COP-22 (2.73 μM) on LPS-induced NO production in Raw264.7 cells was better than EF24 (15.72 μM). COP-22 can also reduce the severity of clinical symptoms of ulcerative colitis, as well as the colonic pathological damage in the DSS-induced mouse model of colitis. However, COP-22 has poor water solubility; therefore, it cannot be used to prepare its solution for intravenous injection.

The liver can sequester most nanoparticles from blood circulation, which becomes the intrinsic advantage of the nanodrug-delivery system (nano-DDS) targeting hepatic diseases [25]. This can increase the targeted accumulation of drugs in the liver, and improve the bioavailability of free drugs, so as to significantly improve the therapeutic effect of ALI. Serum albumin nanocarriers have become one of the most promising carriers, because of their good biocompatibility, low immunogenicity, biodegradability, and good toxicological profiles [26,27,28,29]. Bovine serum albumin (BSA) has been widely studied, because of its high sequence homology (76.52%) with human serum albumin (HSA), wide sources, and low cost [30,31,32,33]. In the present study, COP-22-loaded BSA nanoparticles (22 NPs) were designed, prepared, and characterized. We then analyzed whether 22 NPs could protect against acute liver injury in Lps/D-galN-induced mice. The underlying mechanism of 22 NPs was also investigated.

## 2. Results

### 2.1. Preparation and Characterization of 22 NPs

#### 2.1.1. The Particle Size, Morphology, and Stability

**Figure 2:**
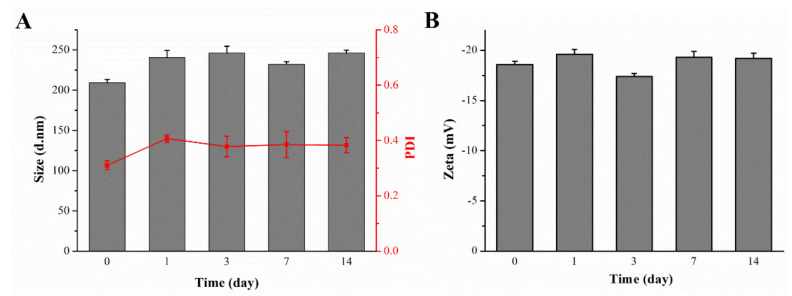
Particle size, PDI changes (**A**), and zeta potential changes (**B**) in 22 NPs. (*n* = 3).

In order to load COP-22 into BSA nanoparticles, a COP-22/E80 complexing agent was prepared first with the egg yolk lecithin E80 (E80), which could improve the lipophilicity and solubility of COP-22. Different preparation conditions of 22 NPs were utilized so as to search the optimized preparation condition. As shown in Table S1, condition 8 was suitable for the preparation of 22 NPs. As shown in Table 1, 22 NPs displayed an average size of 209.2 ± 4.2 nm with PDI of 0.310 ± 0.016, which indicated that the nanoparticles were uniform and in good dispersion condition. Additionally, the zeta potential of 22 NPs was −18.6 ± 0.3 mV, which indicated that the nanoparticles were moderately stable in an aqueous environment. The encapsulation efficiency (EE) of 22 NPs was 99.98%, and the drug-loading content (DLC) was 4.19%. These indicated that 22 NPs could provide sufficient space for accommodating drug molecules and prevent them from leaking. In addition, the stability of 22 NPs was performed at 4 °C for 14 days with size measurement by DLS. Results in Figure 2 revealed that 22 NPs remained stable in an aqueous environment for 14 days as their particle size, PDI, and zeta potential were basically unchanged.

#### 2.1.2. Solid State Characterization

**Figure 3:**
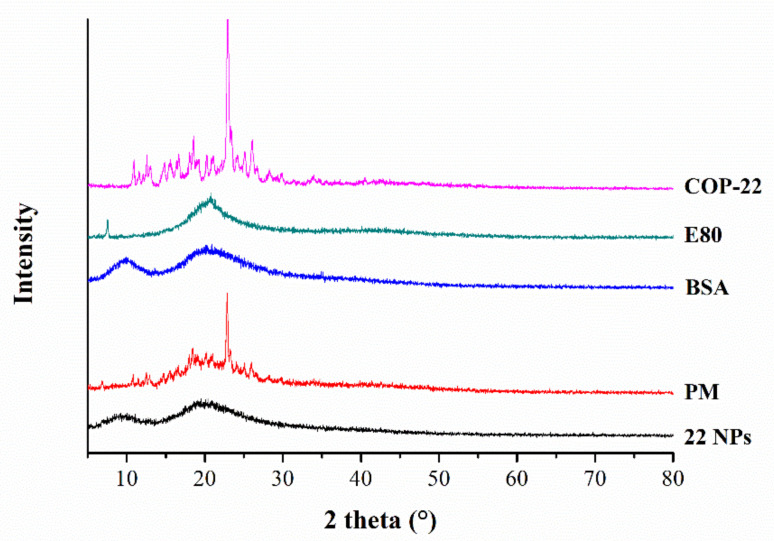
Powder X-ray diffraction patterns of COP-22, E80, BSA, physical mixture (PM) of COP-22/E80/BSA (1/1/1, *w*/*w*/*w*), and 22 NPs.

The XRD diffractograms of free COP-22, free E80, free BSA, physical mixture of COP-22/E80/BSA, and 22 NPs are exhibited in Figure 3. Free COP-22 showed intense diffraction peaks, which indicated the crystalline nature of free COP-22. The physical mixture of COP-22/E80/BSA also showed intense diffraction peaks. In contrast, free E80, free BSA, and 22 NPs did not show any sharp peaks. The 22 NPs did not show any free COP-22′ sharp peaks, which indicated that COP-22 was in an amorphous state in 22 NPs, and further proved that COP-22 was encapsulated within BSA nanoparticles.

#### 2.1.3. Binding Properties of COP-22 with BSA

**Figure 4:**
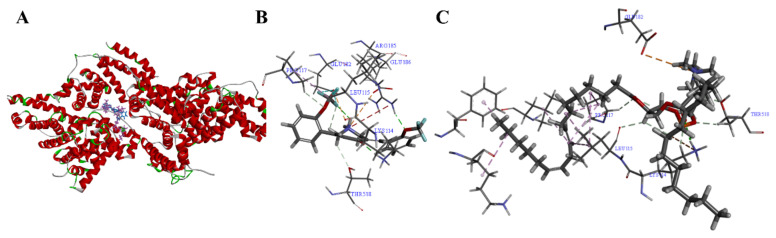
COP-22 and E80 in the binding site of BSA (**A**) and their enlarged pictures (**B**,**C**).

To further clarify the binding properties of COP-22 with BSA and determine their binding sites, molecular docking of COP-22 and E80 with BSA was performed with Discovery Studio 2019 software. As shown in Figure 4 and Figure S1, COP-22 and E80 can interact with the amino acid residues of BSA via conventional hydrogen bonds, carbon hydrogen bonds, halogens, alkyls, attractive charges, etc.. These results indicated that COP-22 could be effectively bound to BSA by interacting with amino acid residues, and could be encapsulated within BSA nanoparticles with high EE% of COP-22. 

### 2.2. In Vitro Study

#### 2.2.1. Inhibitory Effect of 22 NPs on the LPS-Induced NO and ROS Production

**Figure 5:**
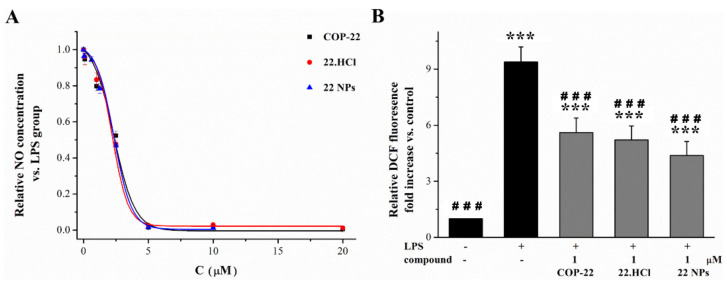
The comparison of COP-22, 22 NPs, and 22 HCl salt on the inhibitor activity of NO (**A**) or ROS (**B**) production. Data were expressed as means ± SD (*n* = 3) from independent experiments. *** *p* < 0.001, compared with the control group. ^###^ *p* < 0.001, compared with the model group.

COP-22 can inhibit the production of NO and ROS in the LPS-induced Raw264.7 macrophage cells [24], which was a classical model for initial screening of anti-inflammatory agents. In order to prepare its solution for intravenous injection, 22 NPs and 22.HCl salt with higher solubility were prepared. In order to verify whether 22 NPs and 22.HCl salt have the same biological activity to inhibit the production of NO and ROS as COP-22, they were tested for their inhibitory effect in the LPS-induced Raw264.7 macrophage cells. As shown in Figure 5A,B, COP-22, 22.HCl salt, and 22 NPs showed similar activity of inhibiting NO and ROS production. 

#### 2.2.2. Effect of Cellular Uptake Activity

**Figure 6:**
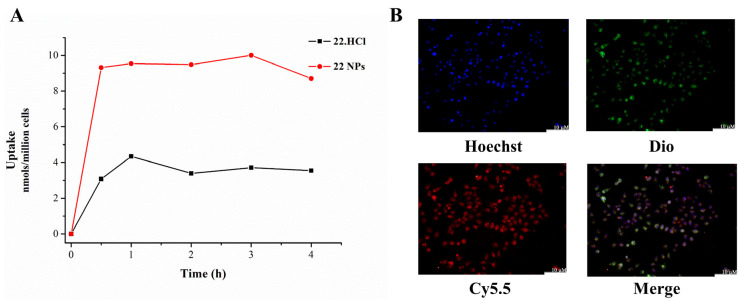
(**A**) Cellular uptake of 22 NPs and 22.HCl salt in Raw264.7 cells. (**B**) The representative fluorescence microscopy images (200×) using L02 cells after 4 h incubation with Cy5.5-COOH labeled 22 NPs. Scale bar: 10 μm.

Next, 22 NPs and 22.HCl salt were examined to compare their cellular uptake ability according to the previously published paper. As shown in Figure 6, 22 NPs displayed a 2–3-fold greater COP-22 accumulation than 22.HCl salt in Raw264.7 cells.

In addition, L02 cells were treated with Hoechst 33342, Dio, and Cy5.5-COOH labeled 22 NPs. The results reflected that 22-NPs could effectively penetrate the cellular membrane to enter the cells.

Through the above cell experiments, it was proved that 22 NPs can effectively enter the cells and play an effective biological activity. Therefore, 22 NPs were further used to study in the mouse model of LPS/D-GalN-induced acute liver injury.

### 2.3. In Vivo Study

#### 2.3.1. Pathological Histology of Liver Tissues

**Figure 7:**
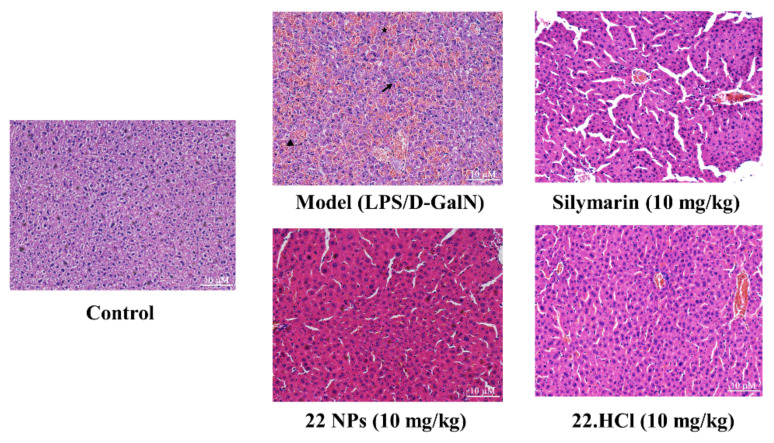
The representative histological microphotographs of H&E-stained liver sections (200×). Scale bar: 10 μm.

LPS and D-GalN are metabolized in the liver and induce liver injury. Histopathological analysis of liver tissue sections using hematoxylin–eosin (HE) staining was conducted to examine liver changes. As shown in Figure 7, the liver tissue of the control group of mice was intact, the cell morphology was normal, and the hepatocytes were arranged in order. In contrast, the model group exhibited infiltration of inflammatory cells (arrow), erythrocyte influx (hemorrhage) (star), and loss of hepatic architecture (arrowhead). Treatment with silymarin, 22.HCl salt, or 22 NPs attenuated LPS/D-GalN-induced acute liver injury, compared with the model group. Histopathological observation directly showed that 22 NPs possess a protective effect against LPS/D-GalN-induced acute liver injury.

#### 2.3.2. Effects of 22 NPs on Liver Function-Related Indexes in Serum of Mice

**Figure 8:**
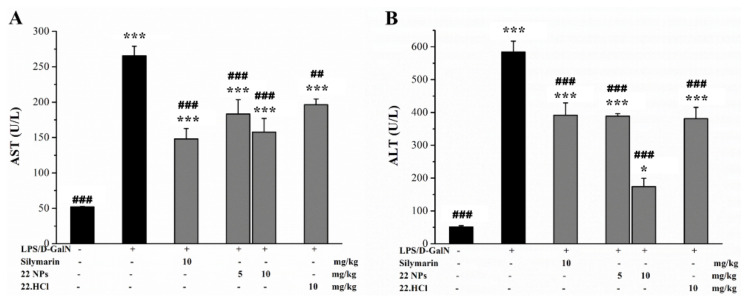
Effects of 22 NPs and 22.HCl salt on the levels of AST (**A**) and ALT (**B**) in serum of mice. Values were mean ± SD (*n* = 8). * *p* < 0.05, *** *p* < 0.001, compared with the control group. ^##^ *p* < 0.01, ^###^ *p* < 0.001, compared with the model group.

Serum ALT and AST are very sensitive to liver injury [34] and have long been used clinically as indicators of liver injury. At 6 h after the injection of LPS/D-GalN, the AST and ALT levels in serum of the model group significantly increased, as compared with the control group (Figure 8). However, after pre-treatment with silymarin, 22.HCl salt, or 22 NPs, the LPS/D-GalN-induced upregulated AST and ALT levels were suppressed. These results above demonstrated that 22.HCl salt and 22 NPs exerted remarkable protective effects on acute liver injury in LPS/D-GalN-treated mice.

#### 2.3.3. The 22 NPs Attenuated LPS/D-GalN-Induced Inflammation in LPS/D-GalN-Induced Mice

In the LPS/D-GalN-induced model, LPS can activate Kuffer cells, and result in the production of pro-inflammatory cytokines, such as tumor necrosis factor-alpha (TNF-α), interleukin-1 (IL-1), and interleukin-6 (IL-6). The nuclear factor kappa B (NF-κB) signaling pathway is an important nuclear transcription factor that activate the transcription of inflammatory factors and the synthesis of many other cytokines [35]. When macrophage cells are stimulated, activated IκB is phosphorylated; thus releasing the NF-κB heterodimers (p65/p50), which then translocate into the nucleus, and further to bind to pro-inflammatory genes.

**Figure 9:**
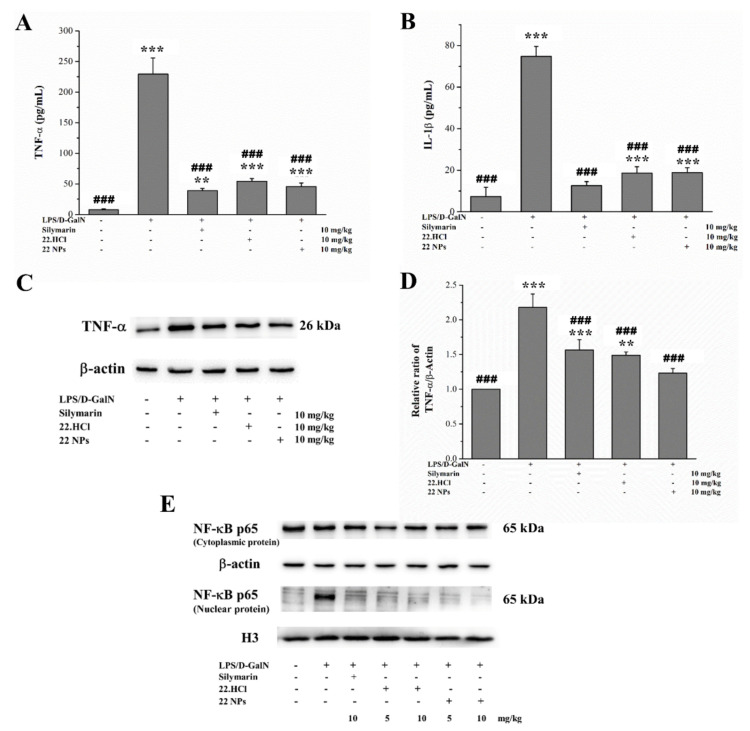
Effects of 22 NPs and 22.HCl salt on inflammation in LPS/D-GalN-induced mice. (**A**,**B**) Levels of serum TNF-α and IL-1β were examined by ELISA. (**C**,**D**) Level of TNF-α in the liver of mice was examined by Western blot analysis. (**E**) The expressions of NF-κB p65 of cytoplasmic and nuclear proteins were assessed by Western blot analysis. Values are mean ± SD (*n* = 8) (**A**,**B**). Data were expressed as means ± SD (*n* = 3) from independent experiments (**D**). ** *p* < 0.01, *** *p* < 0.001, compared with the control group. ^###^ *p* < 0.001, compared with the model group.

As shown in Figure 9A,B, LPS/D-GalN significantly increased serum TNF-α and IL-1β levels in the model group. However, 22 NPs and 22.HCl salt suppressed the increased TNF-α and IL-1β levels. Meanwhile, 22 NPs and 22.HCl salt reversed the increased TNF-α level in liver tissues of LPS/D-GalN-induced mice (Figure 9C,D).

As shown in Figure 9E, the expression of NF-κB p65 in cytoplasmic and nuclear extracts was analyzed. When treated with LPS/D-GalN only, the expression of NF-κB p65 in the nuclei markedly increased. However, the nuclear levels of NF-κB p65 were reduced obviously by 22 NPs and 22.HCl salt treatment. Additionally, 22 NPs and 22.HCl salt possess anti-inflammatory effects similar to that of silymarin in LPS/D-GalN-induced mice. These results above demonstrated that 22.HCl salt and 22 NPs could inhibit liver inflammatory response and protect against LPS/D-GalN-induced liver injury possibly by suppression of NF-κB signaling.

#### 2.3.4. The 22 NPs Attenuated LPS/D-GalN-Induced Oxidative Stress in LPS/D-GalN-Induced Mice

Oxidative stress plays an important role in inflammation, and can cause an imbalance of cellular redox homeostasis, which further aggravate the inflammatory reaction. Antioxidative enzymes can protect hepatocytes from oxidative injury in the liver. Many antioxidative genes are regulated by nuclear factor erythroid 2-related factor2 (Nrf2). Nrf2 is a key transcription factor in the oxidative stress response, and the central regulator of cell antioxidant reduction; and Nrf2-dependent genes and proteins, such as heme oxygenase-1 (HO-1), catalases (CAT), NADPH quinone oxidoreductase 1 (NQO1), etc.. HO-1, a cellular stress protein belonging to the heme oxygenase enzyme system, is a substance with antioxidant activity. CAT are enzymes designed to neutralize the hydrogen peroxide that is formed in vivo by various oxidases and other enzymes. Myeloperoxidase (MPO) is an enzyme of peroxidases found in azurophilic granules of neutrophils. Moreover, malonic dialdehyde (MDA) is an important end product of lipid peroxidation, and an oxidative stress marker. NO is a key inflammatory intermediate.

**Figure 10:**
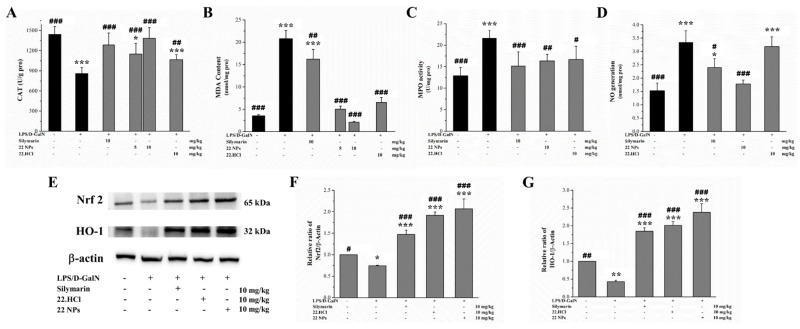
Effects of 22 NPs and 22.HCl salt on oxidative stress in liver tissue in LPS/D-GalN-induced mice. (**A**–**D**) Levels of CAT, MDA, MPO, and NO in the liver were examined. (**E**–**G**) Levels of Nrf-2 and HO-1 in the liver of mice were examined by Western blot analysis. Values are mean ± SD (*n* = 8) (**A**,**B**). Data were expressed as means ± SD (*n* = 3) from independent experiments (**D**). * *p* < 0.05, ** *p* < 0.01, *** *p* < 0.001, compared with the control group. ^#^ *p* < 0.05, ^##^ *p* < 0.01, ^###^ *p* < 0.001, compared with the model group.

As shown in Figure 10A, the level of CAT in the liver of LPS/D-GalN-induced mice in the model group was lower than that in the control group. However, 22 NPs or 22.HCl salt effectively inhibited the decrease. As shown in Figure 10B–D, the levels of MDA, MPO, and NO in the liver of LPS/D-GalN-induced mice in the model group significantly increased relative to those in the control group. When treated with 22 NPs or 22 HCl salt, these levels were significantly decreased as compared to those in the model group.

As shown in Figure 10E–G, the expressions of Nrf2 and HO-1 in the liver of LPS/D-GalN-induced mice were downregulated in the model group. Compared to the model group, 22 NPs and 22.HCl salt treatments upregulated the expressions of Nrf2 and HO-1. These results above suggested that 22 NPs and 22.HCl salt can protect against LPS/D-GalN-induced liver injury in mice by activating Nrf2/HO-1 signaling to resist oxidative stress.

#### 2.3.5. The 22 NPs Alleviated LPS/D-GalN-Induced Hepatic Apoptosis in LPS/D-GalN-Induced Mice

Hepatocyte apoptosis is an important pathological manifestation of acute liver injury and is a hallmark of LPS/D-GalN-induced liver injury of mice. TUNEL staining was used to characterize the apoptotic response and to locate the apoptotic cell in liver sections. Among many apoptosis regulatory genes, the Bcl-2 protein family and caspase family have attracted the most attention. The Bcl-2 family includes two functionally different proteins: antiapoptotic type (such as Bcl-2) and proapoptotic type (such as Bax). The Bcl-2 gene and Bax gene are the most important regulatory genes with opposite functions in the process of apoptosis regulation. Caspase-3 is the most critical apoptosis executing protease in the process of apoptosis.

**Figure 11:**
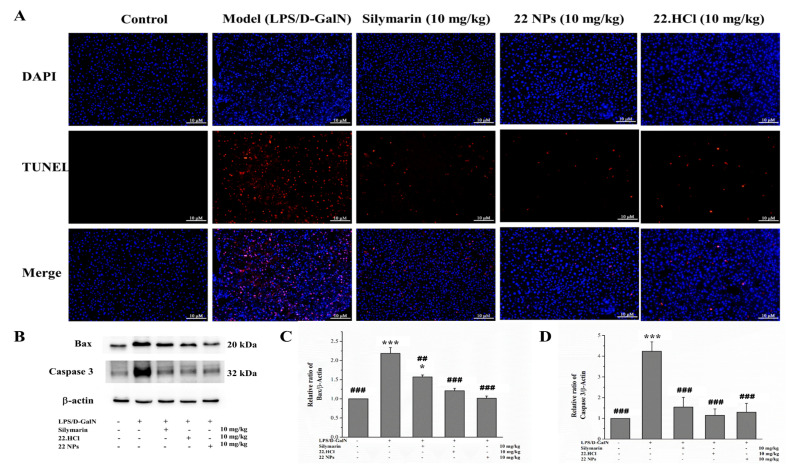
Effect of 22 NPs and 22.HCl salt on apoptosis in LPS/D-GalN-induced mice. (**A**) The representative fluorescence microscopy images (200×) of TUNEL staining. (**B**–**D**) The expressions of Bax and caspase-3 proteins were assessed by Western blot analysis. Data were expressed as means ± SD (*n* = 3) from independent experiments (C and D). * *p* < 0.05, *** *p* < 0.001, compared with the control group. ^##^ *p* < 0.01, ^###^ *p* < 0.001, compared with the model group. Scale bar: 10 μm.

As shown in Figure 11A, the liver sections were stained with TUNEL (red) and DAPI (blue). There were no TUNEL-positive hepatocytes observed in the control group. However, a large number of TUNEL-positive hepatocytes were observed in the model group. When pretreated with 22 NPs or 22.HCl salt, the number of TUNEL-positive hepatocytes was significantly decreased.

As shown in Figure 11B–D, Bax and caspase-3 levels were significantly upregulated in the LPS/D-GalN group, compared to those observed in the control group. The groups pretreated with 22 NPs or 22.HCl salt showed downregulated expression of Bax and caspase 3 compared to the model group. Additionally, 22 NPs and 22.HCl salt possesses a protective effect more than that of silymarin against LPS/D-GalN-induced liver injury. These results above demonstrate that the protective effects of 22.HCl salt and 22 NPs against LPS/D-GalN-induced liver injury could be related to the inhibitory ability of hepatocellular apoptosis through inhibiting the upregulation of Bax and caspase-3.

In LPS/D-GalN-induced acute liver injury of mice, LPS can activate Kuffer cells, and NF-κB is translocated into the nucleus, where it initiates transcription of multifarious inflammatory cytokines, such as TNF-α, IL-1, IL-6, etc. TNF-α is the most important pleiotropic cytokines, which can trigger an inflammatory cascade to induce other cytokines [36,37]. TNF-α binds to its receptor on the membrane of hepatocytes; thus, activating caspase-3 and inducing apoptosis [38].

Nrf2 is an important regulatory factor that regulates cellular defense against oxidative stress. Activation of Nrf2 also confers an anti-inflammatory effect, by blocking NF-κB transcription and inhibiting inflammatory cytokines [39]. Activated Nrf2 can downregulate NF-κB signaling and activated NF-κB can also downregulate Nrf2 signaling. This is because p65 binds to keap1 and inhibits the dissociation of Nrf2 from keap1, which acts as a bridge between the NF-κB signal pathway and Nrf2 signal. This relationship between Nrf2 and NF-κB is also reflected in this study. In LPS/D-GalN-induced acute liver injury of mice, NF-κB signaling was activated and Nrf2 signaling was inhibited; while, when pretreated with 22 NPs or 22 HCl salt, NF-κB signaling was inhibited and Nrf2 signaling was activated.

#### 2.3.6. Biodistribution Study

**Figure 12:**
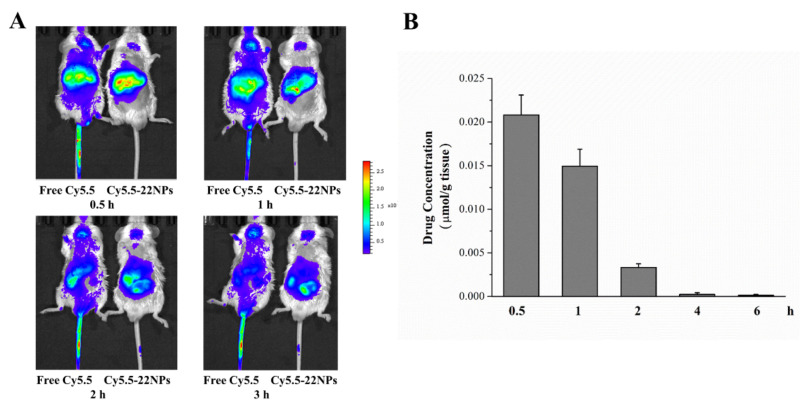
In vivo tissue biodistribution of COP-22. (**A**) In vivo images of mice with Cy5.5-labeled 22 NPs at different times by IVIS. (**B**) COP-22 concentration in liver tissues was determined at different times by HPLC. Data were expressed as means ± SD (*n* = 3) from independent experiments.

Animal fluorescence imaging was performed by the intravenous injection of Cy5.5-22NPs and free Cy5.5 (Figure 12A). At 0.5 h, the fluorescence signal intensity of the liver was significant, and the fluorescence signal intensity of the Cy5.5-22 NPs group was higher than in the free Cy5.5 group. In other words, 22 NPs can quickly accumulate in liver tissue. In contrast, the fluorescence signal intensity of the liver decreased at 1 h. At 2 and 3 h, the fluorescence intensity signal moved down, suggesting that Cy5.5-22 NPs was gradually metabolized out of the body. In addition, compared with the Cy5.5-22 NPs group, the fluorescence of the free Cy5.5 group was distributed in the whole body of mice throughout the experiment. These results suggested that Cy5.5-22 NPs could be passively targeted to accumulate in the liver through a retention effect, and then metabolized; in contrast, free Cy5.5 needed to be absorbed and metabolized more slowly.

The COP-22 concentration in liver tissues at different times was also determined by high performance liquid chromatography (Figure 12B). For liver tissue samples injected with 22NPs, the concentration of COP-22 was higher at 0.5 h. Over time, the concentration of COP-22 decreased gradually, and the change trend was consistent with the change in fluorescence intensity in vivo imaging. For liver tissue samples injected with 22.HCl salt, the concentration of COP-22 was lower than the detection limit. Therefore, the tissue concentration was increased 6-fold for detection. The concentration of COP-22 at 0.5 h could be determined, but the concentration is very low (0.88 nmol/g issue). Additionally, the concentration of COP-22 at other time points could not be detected even if it was concentrated 6-fold. By comparison, 22 NPs are more easily absorbed than 22.HCl salt in the liver tissue.

## 3. Conclusions

In conclusion, this study demonstrated that 22 NPs exhibited effective hepatoprotective effects by inhibiting inflammation, oxidative stress, and apoptosis on LPS/D-GalN-induced acute liver injury. The anti-inflammatory activity of 22 NPs was largely related to the regulation of the NF-κB signaling pathways; the antioxidant activity of 22 NPs was related to the regulation of the Nrf2 signaling pathway; and the apoptosis activity of 22 NPs was related to the mitochondrial pathway, which involved the Bcl-2 family and Caspase-3 protein. Three cellular pathways were interrelated and affect each otherMoreover, compared with 22.HCl salt, 22 NPs could be passively targeted to accumulate in the liver through the retention effect.

## 4. Materials and Methods

### 4.1. Materials

2-Trifluoromethoxy-substituted 4-piperidione-containing mono-carbonyl curcumin derivative COP-22 was synthesized from 2-trifluoromethoxy-substituted benzadehyde (Energy Chemical, W3100580250, Shanghai, China) and 4-piperidione (Energy Chemical, E011401, Shanghai, China) in ethanol, according to our previously published paper [40]. Its salt was obtained from an anhydrous ethyl acetate solution containing hydrogen chloride. All the other chemicals and solvents were of the highest grade commercially available and used without further purification.

### 4.2. Preparation of 22 NPs

Previous reported similar method was utilized to prepare 22 NPs [41]. First, 10 mg of COP-22 and 50 mg of the egg yolk lecithin E80 (E80) (Yuanye, S30871, Shanghai, China) in 2 mL of methanol were stirred for 1 h at 40 °C to gain the clear solution. After stirring, methanol was removed using the rotary evaporator, and then, COP-22/E80 complexing agent was formed. This COP-22/E80 complexing agent was re-dissolved in 1 mL of methylene chloride, as the organic phase. Next, 200 mg of BSA (Beyotime, ST023, Shanghai, China) was dissolved in 10 mL ddH_2_O, as the aqueous phase. Additionally, the organic phase was slowly added dropwise to the aqueous phase under ultrasonic waves, and the mixture was sonicated for another five minutes. Finally, the COP-22-loaded BSA nanoparticles (22 NPs) were constructed after the removal of organic solvent.

### 4.3. Characteristics of 22 NPs

#### 4.3.1. Particle Size, Zeta Potential, and PDI

The particle size, zeta potential, and PDI of 22 NPs were analyzed via dynamic light scattering (DLS, Zetasizer Nano ZSP, Malvern, UK). An integrated 4 mV He-Ne laser (λ = 633 nm) and scattering angle θ = 173° backscattering detection were set for DLS detection. The experiments were performed in triplicate.

#### 4.3.2. Encapsulation Efficiency (EE) and Drug-Loading Content (DLC)

**Figure 13:**
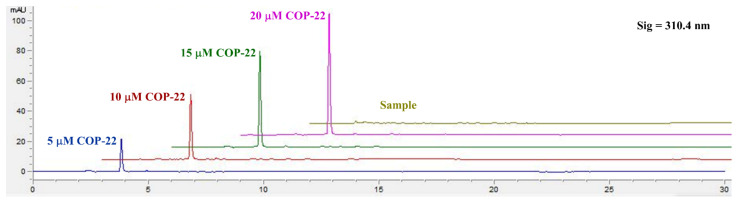
The representative HPLC chromatogram of standard curves and ultra-filtrate of 22 NPs for the evaluation of encapsulation efficiency.

For EE, 22 NPs were ultra-filtrated by Milipore Ultra centrifugal filters (NMWL = 10 kDa). The filtrate was used for determination. For DLC, 22 NPs were treated with vacuum freeze-drying, and weighed. The EE (%) and DLC (%) for 22 NPs were detected via high performance liquid chromatography (HPLC, Agilent Technologies 1260 Infinity, Germany) by analyzing COP-22. For HPLC detection, methanol/H_2_O (90/10, *v*/*v*) was used as the mobile phase at the flow rate of 1 mL/min. The standard curves were determined using 20 μL of 20, 15, 10, and 5 μM COP-22 in methanol/H_2_O (1/1, *v*/*v*) solution (Figure 13). (COP-22, 3.812 min, 310.4 nm, S (mAU∗S) = 5.414 + 21.8824 C (μM)).

EE (%) and DLC (%) were calculated as follows.

EE (%) = (Total amount of the bound drug in the NPs)/(Total feed amount of the drug) × 100%
= (Total feed amount of the drug—the amount of free drug)/(Total feed amount of the drug) × 100%
DLC (%)
= (Total amount of the bound drug in the NPs)/(Total weight of the NPs) × 100%
= (Total feed amount of the drug—the amount of free drug)/(Total weight of the NPs) × 100%.

#### 4.3.3. X-ray Diffraction (XRD)

The crystalline state of COP-22 in the 22 NPs was investigated using an X-ray powder diffractometer (DX-2700B, Haoyuan, Dandong, China) operated at 40 kV and 40 mA with a Cu Kα radiation detector (λ = 1.54 Å). Powder of free COP-22, free E80, free BSA, physical mixture of COP-22/E80/BSA, and 22 NPs were added into the sample holder. The XRD patterns were recorded at a scanning range of 3–80° and a scanning rate of 6° per minute.

### 4.4. Molecular Docking Studies

Molecular docking studies was conducted with discovery studio 2019 software. Crystal structure of bovine serum albumin (BSA) was downloaded from RCSB PDB database (http://www.rcsb.org, accessed on 10 March 2022) with PDBID 4F5S. The combined spherical areas of BSA were x = 48.7282, y = 22.5842, z = 97.654, radius = 32.3. The molecular docking module (CDocker) in the DS 2019 software was used for docking, and the simulation with the highest CDOCKER score was analyzed. DS 2019 and ChemBioDraw Ultra 14.0 were used to construct the target compound’s structural formula.

### 4.5. Cell Culture

L02 and Raw264.7 cells were purchased from the Shanghai Institute of Biochemistry and Cell Biology, Chinese Academy of Sciences. L02 and Raw264.7 cells were cultured in 1640 (Hyclone, SSH30809.01, American) and DMEM (Hyclone, SSH30243.01, American), respectively, supplemented with 10% fetal bovine serum (Gibco, C0232, American), and 100 U/mL penicillin/streptomycin (Beyotime, C0222, Shanghai, China) in a humidified 5% CO_2_ atmosphere at 37 °C (Thermo Scientific HERAceu150i, American). The cells were resuscitated and subcultured for three passages before use.

### 4.6. Measurement of NO Production

Nitrite accumulated in the culture medium was measured by Griess reagent. First, 100 μL Raw264.7 cells were seeded at 1 × 10^6^ cells/mL in 96-well plates (Costar) for 24 h and then incubated with LPS (Sigma, L4391, Shanghai, China) (1 μg/mL) and different concentrations (20, 10, 5, 2.5, and 1.25 μM) of the test compounds for 24 h. After incubation, 75 μL of cell supernatant were distributed in a 96-well plate and then equal volumes of the fresh Griess reaction solutions (0.1% *N*-(1-naphthyl)-ethylenediamine dihydro-chloride (Sigma, 222488, Shanghai, China) and 1% sulfanilamide (Sigma, PHR1184, Shanghai, China) in 2.5% phosphoric acid (Sigma, 695017, Shanghai, China) were added. After 5 min of incubation at RT, the absorbance at 540 nm was measured on a multiwall plate reader (Biotek Synergy H1 multi format microplate readers, American). Assays were performed in triplicate and repeated three times.

### 4.7. Determination of Intracellular ROS Levels

Intracellular ROS levels were determined using the redoxsensitive fluorescent probe dichlorofluorescein diacetate (DCFH-DA) (Beyotime, S0033S-1, Shanghai, China). Raw264.7 cells were seeded at 3 × 10^6^ cells/well in 6-well plates and incubated for 24 h. After treatment with LPS (1 μg/mL) and the test compounds (1 μM) for 24 h, the cells were collected, and washed twice with phosphate buffer solution (PBS, 10 mM, pH 7.4). Then, they were resuspended in PBS and incubated with 3 μM DCFH-DA for 30 min at 37 °C in the dark. The cells were collected, washed with PBS, and analyzed immediately for DCFH-DA fluorescence intensity (excitation wavelength 488 nm and emission wavelength of 530 nm) with a FACSCanto flow cytometer (Millipore Guava easyCyte 8HT benchtop flow cytometer, American). A total of 10,000 events were acquired per sample. Three independent experiments were carried out.

### 4.8. Cellular Uptake Activity

Raw264.7 cells were seeded at 3 × 10^6^ cells/well (2 mL) in 6-well plates and incubated for 24 h. After 0.5, 1, 2, 3, or 4 h of treatment with the test compounds 22.HCl salt and 22 NPs (20 μM), the cells were rapidly washed twice with 1 mL ice-cold PBS. They were then extracted with ice-cold methanol (800 μL/well) at 4 °C overnight. The suspension was centrifuged for 5 min at 10,000 rpm and 4 °C, and the supernatant (200 μL) was determined with using a multiwall plate reader (Biotek Synergy H1 multi format microplate readers, American).

For fluorescence microscope observation of cellular uptake, 2 mL L02 cells were seeded onto sterile glass coverslips placed in 6-well plates at a density of 1 × 10^5^ cells/mL. Then, 22 NPs (22 1 mg/mL, 2.2 mM) were labeled by Cy5.5-COOH (0.2 mg/mL, 0.323 mM) (Macklin, C854973, Shanghai, China), and added into the 6-well plate, which was pre-cultured for 4 h, at an equivalent concentration of COP-22 20 μM and Cy5.5 (2.9 μM). The medium in the wells was discarded after incubation for 4 h, followed by washing with PBS three times. Then, the cells were fixed with 4% paraformaldehyde fix solution (Beyotime, P0099, Shanghai, China) for 15 min at room temperature, followed by washing with PBS three times. Hoechst 33342 (Beyotime, C1022, Shanghai, China) and Dio (Beyotime, C1038, Shanghai, China) were applied to stain the nucleus and membrane of the cells for 10 min at room temperature, respectively. After washing with PBS, the coverslips were mounted onto glass slides using an anti-fade mounting medium (Beyotime, P0126, Shanghai, China). Photographs of the stained samples were taken using an Olympus microscope (BX53 + DP80) (Japan).

### 4.9. Animals

Balb/c mice (8-week-old, male, 18–20 g) were purchased from Pengyue Experimental Animal Breeding Co. LTD (Jinan, Shandong, China). Mice were housed on a 12 h dark/light cycle at a constant temperature of 23 ± 2 °C with free access to a standard diet and water. All animal studies were conducted under the National Institute Guide for the Care and Use of Laboratory Animals. Experimental protocols were approved by the Ethics Committee of Liaocheng University.

### 4.10. Induction of Acute Hepatitis in Mice and Treatment

After 7 days of the adaptation period, the animals were randomly assigned into five groups (*n* = 8 per group): control group, model group, silymarin group, 22 NPs group, and COP-22 group. Silymarin (10 mg/kg, dissolved in 30% N,N-dimethylacetamide *(DMAc)* of saline solution) (Macklin, S873807, Shanghai, China), 22 NPs (22, 5, or 10 mg/kg), and 22.HCl salt (5 or 10 mg/kg, dissolved in 30% DMAc of saline solution) were administered intravenously before 1 h of treatment with 1 mg/kg LPS and 700 mg/kg D-GalN (Beyotime, ST1213, Shanghai, China) by intraperitoneal injection. For the model group, only treatment with LPS and D-GalN occurred. All mice were sacrificed 6 h after the LPS/D-GalN injection, and both blood samples and liver tissues were collected for further analysis.

### 4.11. Measurement of the Levels of AST and ALT

The serum levels of AST and ALT were used as biochemical markers for acute hepatitis. Blood samples were centrifuged at 1000 g at 4 °C for 15 min. The serum was separated, and serum AST (E2023) and ALT (E2021) activities were measured by using commercial kits from Applygen Technologies Inc. (Beijing, China).

### 4.12. Cytokine Determination

The serum levels of cytokines (IL-1β and TNF-α) were determined using the ELISA method. The amounts of IL-1β and TNF-α in the serum were determined using a mouse IL-1 beta ELISA Kit (RayBiotech, ELM-IL 1b, American) and a mouse TNF-alpha ELISA Kit (RayBiotech, ELM-TNFa, American), following the manufacturer’s instructions.

### 4.13. NO Assay of Liver Tissues

The frozen livers were homogenized with 5 times volume PBS (10 mM, pH 7.4) containing proteinase inhibitors (Beyotime, P1010, Shanghai, China). The supernatants were obtained following centrifugation at 15,000 g at 4 °C for 20 min. The protein concentration of the supernatant was measured using the Enhanced BCA Protein Assay Kit (Beyotime, P0010, Shanghai, China). The nitrite content in the supernatant was measured using the Griess reagent. The concentration of NO was calculated by extrapolating a NaNO_2_ standard curve. The amounts of NO were expressed as nmol/mg tissue protein (nmol/mg).

### 4.14. MPO Assay

A total of 40 μL of the supernatant of livers were added to 60 μL PBS (50 mM, pH 6.0), 0.0005% *o*-dianisidine dihydrochloride (Alfa Aesar, A17175, Shanghai, China), and 0.1% hydrogen peroxide. The rate of absorbance changes at 460 nm was measured. One unit of MPO activity is equal to 5.65 × 10^−3^ changes in absorbance at 25 °C. The activity of MPO was expressed as units/mg tissue protein (U/mg pro).

### 4.15. MDA Assay

The amounts of MDA in the supernatants were determined using the Lipid Peroxidation MDA Assay Kit (Beyotime, S0131S, Shanghai, China) as per the manufacturer’s instructions. The content of MDA was expressed as nmol MDA/mg tissue protein (nmol/mg pro).

### 4.16. CAT Assay

CAT activity was measured by analyzing the rate at which it caused the decomposition of H_2_O_2_ at 240 nm using the Catalase Assay Kit (Ultraviolet) (Nanjing Jiancheng Bioengineering Institute, a007-2-1, Nanjing, China). The activity of CAT is expressed as units/g tissue protein (U/g pro).

### 4.17. Histopathology Analysis

Liver tissues were fixed in buffered formalin (Beyotime, P0099, Shanghai, China), embedded in paraffin, and then sectioned. The sections were stained with hematoxylin and eosin (H&E) (Beyotime, C0105, Shanghai, China), and photographed using an Olympus microscope (BX53 + DP80, Japan).

### 4.18. TUNEL Assay

Terminal deoxynucleotidyl transferase (TDT)-mediated dUTP nick-end labeling (TUNEL) assay was performed to detect apoptosis in the liver tissues of mice by using a one-step TUNEL apoptosis assay kit (Beyotime, C1089, Shanghai, China) as per the manufacturer’s protocol. The sections were counterstained with DAPI (Beyotime, C1002, Shanghai, China), and photographed using an Olympus microscope (BX53 + DP80, Japan).

### 4.19. In Vivo Pharmacokinetics and Biodistribution

**Figure 14:**
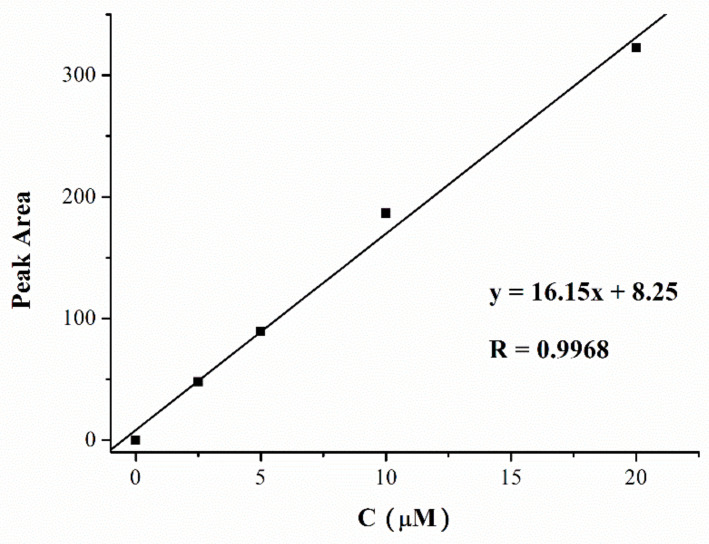
The standard curves of COP-22 in homogenate of liver.

Balb/c mice were administered a single injection of 22.HCl salt or 22 NPs at a dose of 10 mg COP-22/kg, and sacrificed for liver tissue collection at 0, 0.5, 1, 2, 4, 6 h, respectively. Then, 133 mg tissues were placed into 400 μL saline solution following homogenizing using a TissueMaster™ Handheld Homogenizer (Beyotime, E6600, Shanghai, China). A total of 400 μL ethyl acetate was added into the homogenate, vortex mixed, centrifuged, and the ethyl acetate layer was separated. The extraction was repeated three times, and the ethyl acetate solution was combined. The solution was evaporated under reduced pressure, and the 200 μL methanol was added. After filtration, COP-22 concentrations were determined with high performance liquid chromatography (HPLC, Agilent Technologies 1260 Infinity, Waldbronn, Germany). The standard curve was shown in the Figure 14. For samples whose content was lower than the detection limit, the tissue concentration was increased 6-fold for detection.

Cy5.5-COOH dye (0.2 mg/mL, 0.323 mM) (Macklin, C854973, Shanghai, Chian) was used to label the 22 NPs in vivo for the IVIS Lumina LT Series III (PerkinElmer, American). A total of 100 μL 22 NPs or free Cy5.5-COOH dye was administered intravenously into each mouse. Biofluorescent images were obtained using the IVIS imaging system at 0, 0.5, 1, 2, and 3 h.

### 4.20. Western Blot Analysis

The liver tissues were lysed with Radio Immunoprecipitation Assay (RIPA) buffer (Beyotime, P0013B, Shanghai, China) or Nuclear Extraction Kits (Beyotime, P0028, Shanghai, China) supplemented with Protease Inhibitors (Beyotime, P1011, Shanghai, China). Then, Western blot was carried out as the general process. Primary antibodies: NF-κB (Cell Signaling Technology, 8242S, 1:1000, American), TNF-α (Wanleibio, WL01581, 1:1000), Nrf2 (Wanleibio, WL02135, 1:500, Shenyang, China), HO-1 (Wanleibio, WL02400, 1:500, Shenyang, China), Caspase-3 (Santa Cruz Biotechnology, sc-56053, 1:500, American), Bax (Cell Signaling Technology, 2772S, 1:1000, American), β-actin (Cell Signaling Technology, 4970S, 1:1000, American), and H3 (Proteintech, 17168-1-AP, 1:1000, American). Secondary antibodies: HRP-labeled goat anti-rabbit IgG(H + L) (Beyotime, A0208, Shanghai, China) and HRP-labeled goat anti-mouse IgG(H + L) (Beyotime, A0216, Shanghai, China).

### 4.21. Statistical Analysis

The data were presented as the means ± standard deviation (SD) and analyzed using a one-way ANOVA to determine any significant differences. *p* < 0.05 was considered as statistically significant.

## Figures and Tables

**Table 1:** The particle size, PDI, zeta, EE (%), and DLC (%) of 22 NPs.

NPs	Size (d.nm)	PDI	Zeta (mV)	EE (%)	DLC (%)
**22 NPs**	209.2 ± 4.2	0.310 ± 0.016	−18.6 ± 0.3	99.98 ± 0.01	4.19 ± 0.05

## Supplementary Materials

The following are available online at https://www.mdpi.com/article/10.3390/ijms23094903/s1.
