# Supplementary material for: Hepatoprotective Effects of Albumin-Encapsulated Nanoparticles of a Curcumin Derivative COP-22 against Lipopolysaccharide/D-Galactosamine-Induced Acute Liver Injury in Mice
Source: Int J Mol Sci. 2022 Apr 28;23(9):4903. doi: 10.3390/ijms23094903 (PMC9102161; doi:10.3390/ijms23094903)
Supplement: Supplementary file 1 [file ijms-23-04903-s001.zip › ijms-1654644-supplementary.pdf]

Hepatoprotective effects of albumin-encapsulated nanoparticles of a curcumin derivative COP-22 against Lipopolysaccharide/D-Galactosamine-induced acute liver injury in mice

Wenwen Mu, Qi Wang, Mingxia Jia, Sijia Dong, Sijie Li, Jie Yang*, and Guoyun Liu*

School of Pharmaceutical Sciences, Liaocheng University, 1 Hunan Street, Liaocheng, Shandong 252059, China

Supporting Information

* Corresponding author

Fax: (+86) 15063505132

E-mail: yangjie1110@163.com; guoyunliu@126.com

Table S1. The particle size and the zeta potential of 22 NPs prepared by different conditions.

Condition	$C_{COP\text{-}22}$ (mg/mL)	C_{BSA} (%)	$t_{ultrasonic}$ (min)	particle size (d.nm)	Zeta (mV)
1	2	2	30	450.3±41.2	
2	1	2	30	256.6±27.8	
3	1	1	30	539.0±29.8	
4	1	2	40	504.9±22.6	
5	1	2	20	261.7±13.5	1.97±0.06
6	1	2	15	290.8±58.6	-2.46±0.31
7	1	2	10	218.3±11.1	-1.06±0.09
8	1	2	5	209.2±4.2	-18.6±0.3
9	1	2	0	418.4±78.0	-0.55±0.22

Condition 8 is suitable for preparing the 22 NPs, which affords particles with minimum size of 209.2±4.2 nm, and high zeta potential of -18.6±0.3 mV.

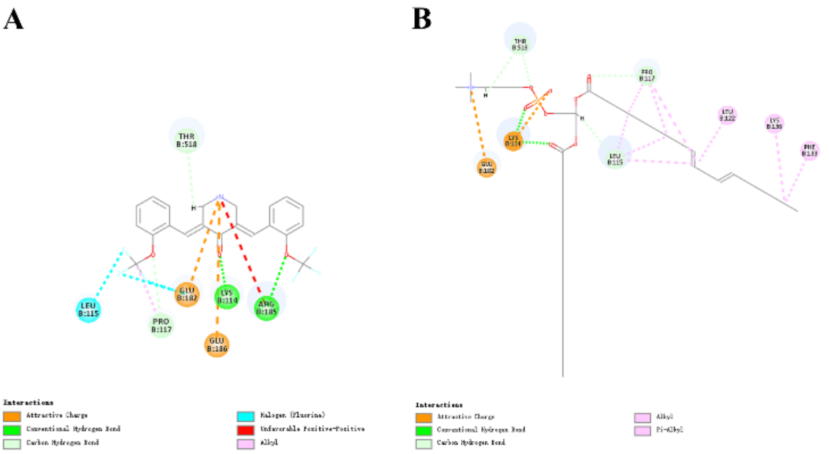

Figure S1. COP-22 and E80 in the binding site of BSA.
